# Supplementary material for: Constituents of the Stem Bark of Trichilia monadelpha (Thonn.) J. J. De Wilde (Meliaceae) and Their Antibacterial and Antiplasmodial Activities
Source: Metabolites. 2023 Feb 17;13(2):298. doi: 10.3390/metabo13020298 (PMC9966138; doi:10.3390/metabo13020298)
Supplement: Supplementary file 1 [file metabolites-13-00298-s001.zip › metabolites-2158909-supplementary.pdf]

Supplementary material

Constituents of the stems bark of *Trichilia monadelpha* and their antibacterial and antiplasmodial activities

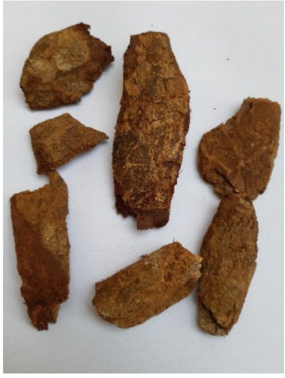

Contents of supplementary material

Figure S1. ^{1}H NMR spectrum of compound **1**

Figure S2. ^{13}C NMR spectrum of compound **1**

Figure S3. DEPT 135° spectra of compound **1**

Figure S4. HMBC spectrum of compound **1**

Figure S5. HSQC spectrum of compound **1**

Figure S6. ^{1}H-^{1}H COSY spectrum of compound **1**

Figure S7. ^{1}H-^{1}H NOESY spectrum of compound **1**

Figure S8. HRESIMS spectrum of $[M+Na]^+$ ion of compound **1**

Figure S9. ^{1}H NMR spectrum of compound **2**

Figure S10. ^{13}C NMR spectrum of compound **2**

Figure S11. DEPT 135° spectra of compound **2**

Figure S12. HMBC spectrum of compound **2**

Figure S13. HSQC spectrum of compound **2**

Figure S14. ^{1}H-^{1}H COSY spectrum of compound **2**

Figure S15. NOESY spectrum of compound **2**

Figure S16. HRESIMS spectrum of $[M+H]^+$ ion of compound **2**

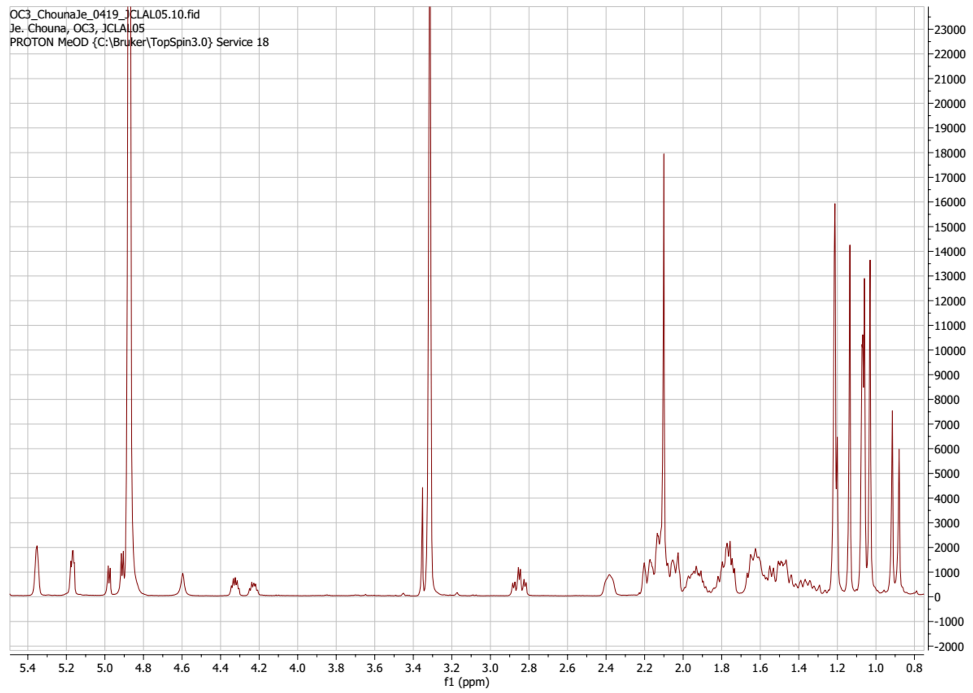

Figure S1. ^{1}H NMR spectrum of compound **1**

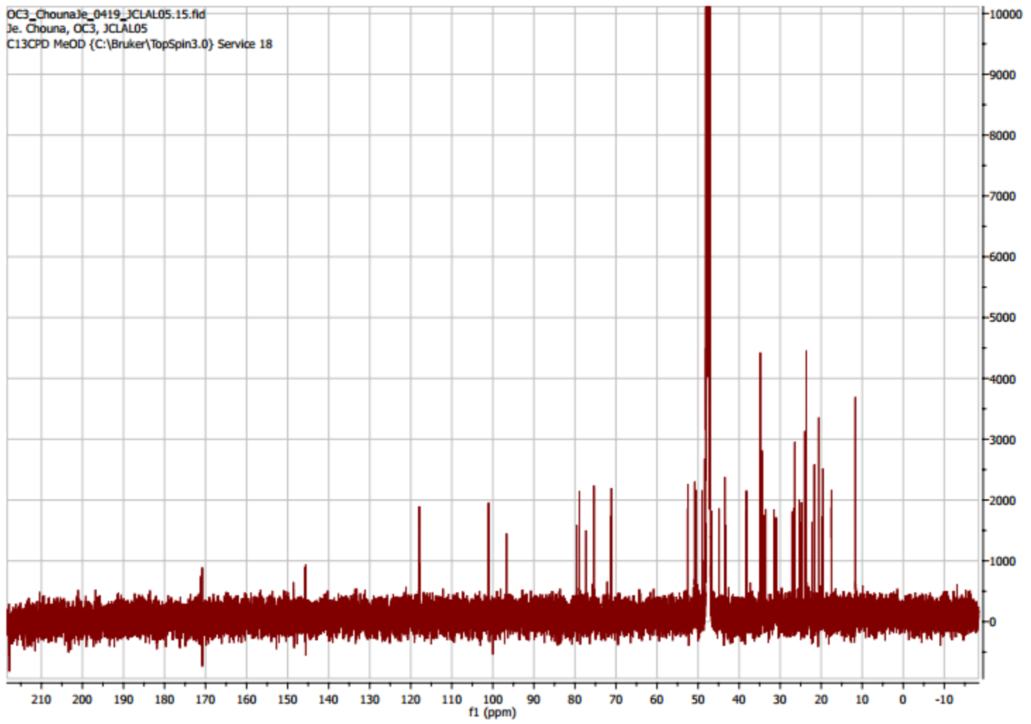

Figure S2. ^{13}C NMR spectrum of compound **1**

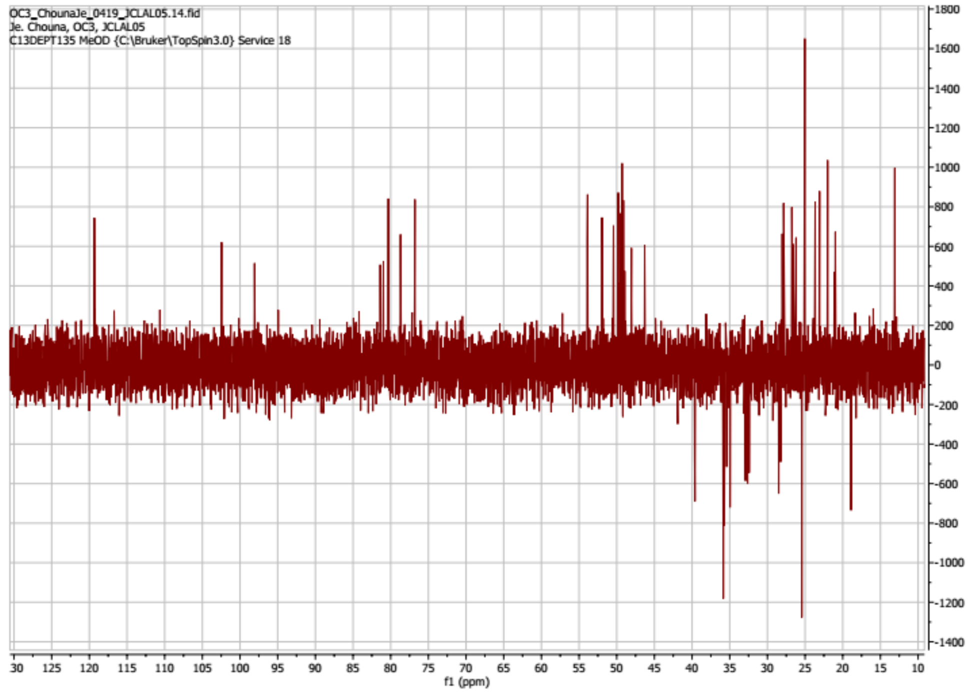

Figure S3. DEPT 135° spectra of compound **1**

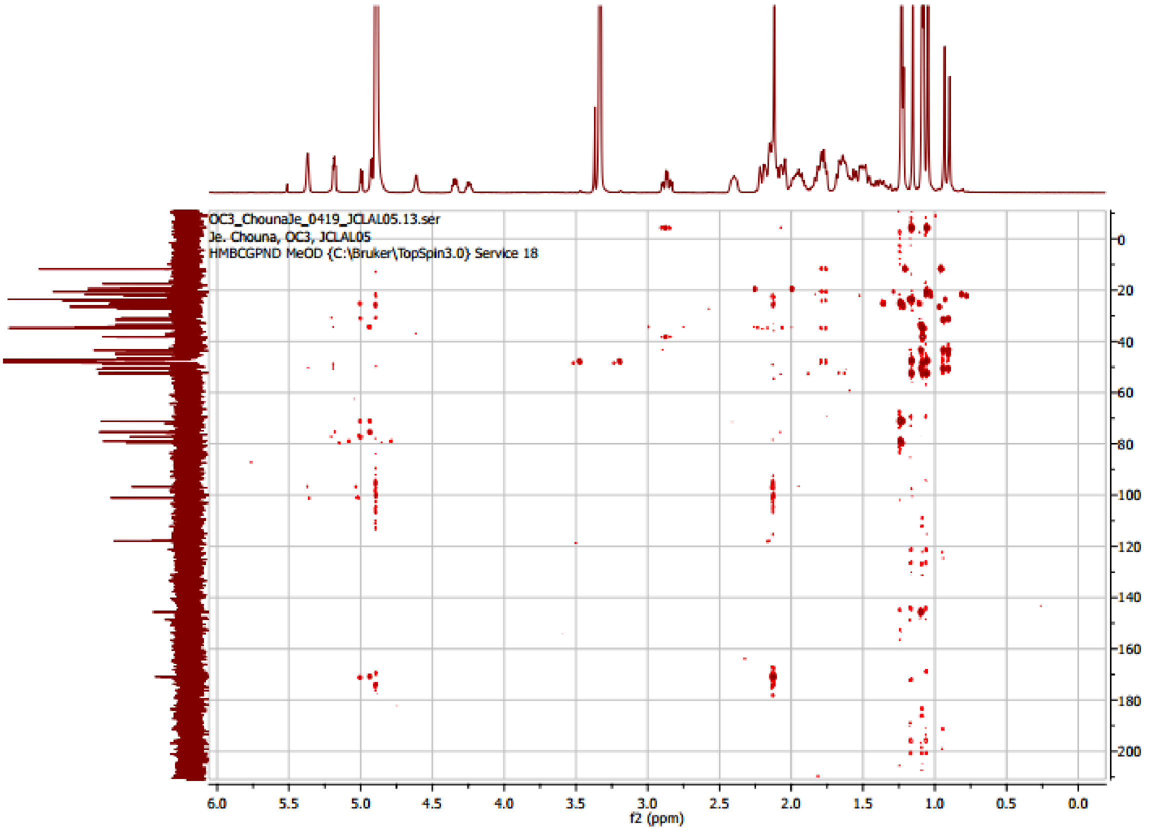

Figure S4. HMBC spectrum of compound **1**

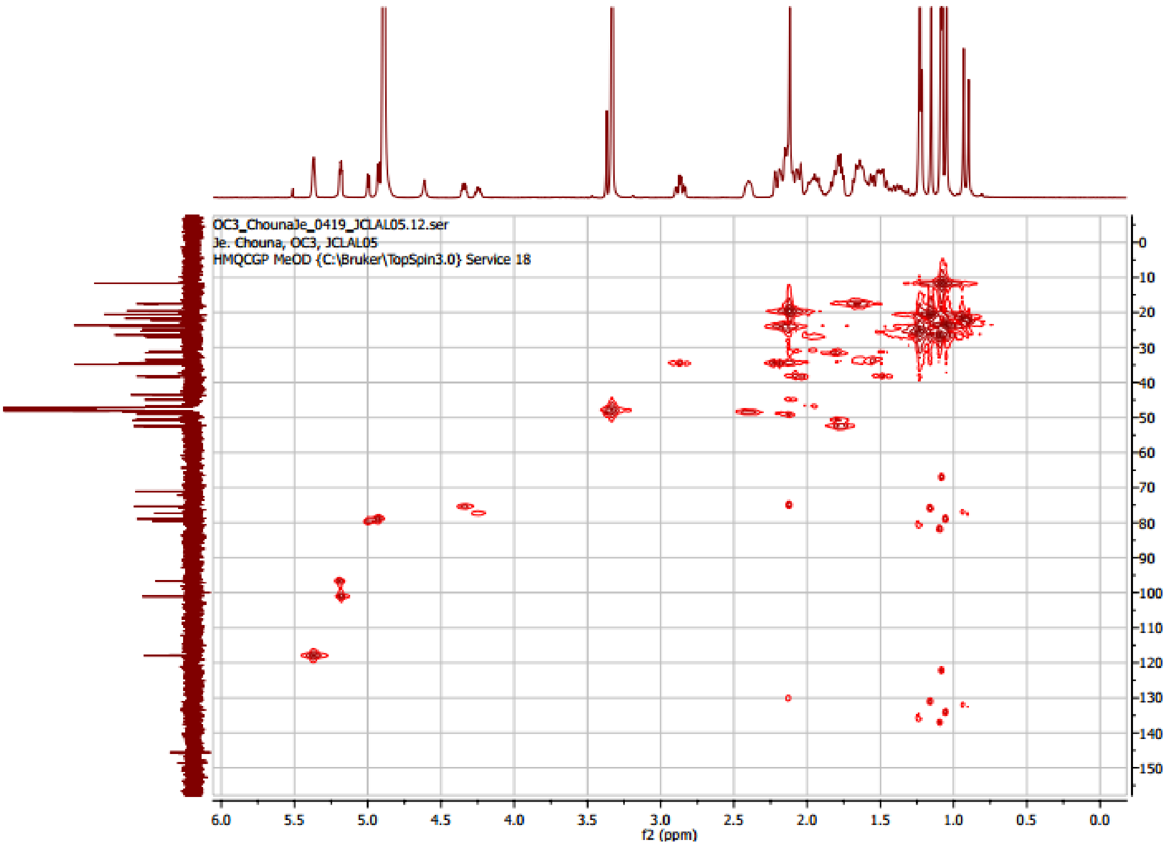

Figure S5. HSQC spectrum of compound **1**

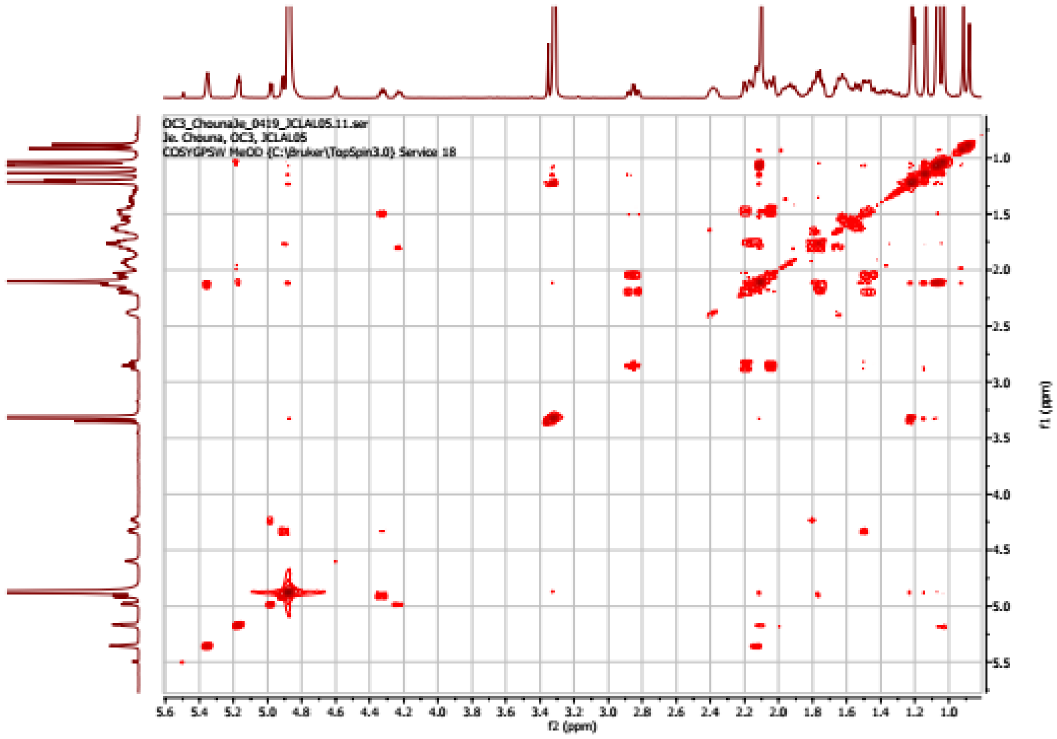

Figure S6. ^{1}H-^{1}H COSY spectrum of compound **1**

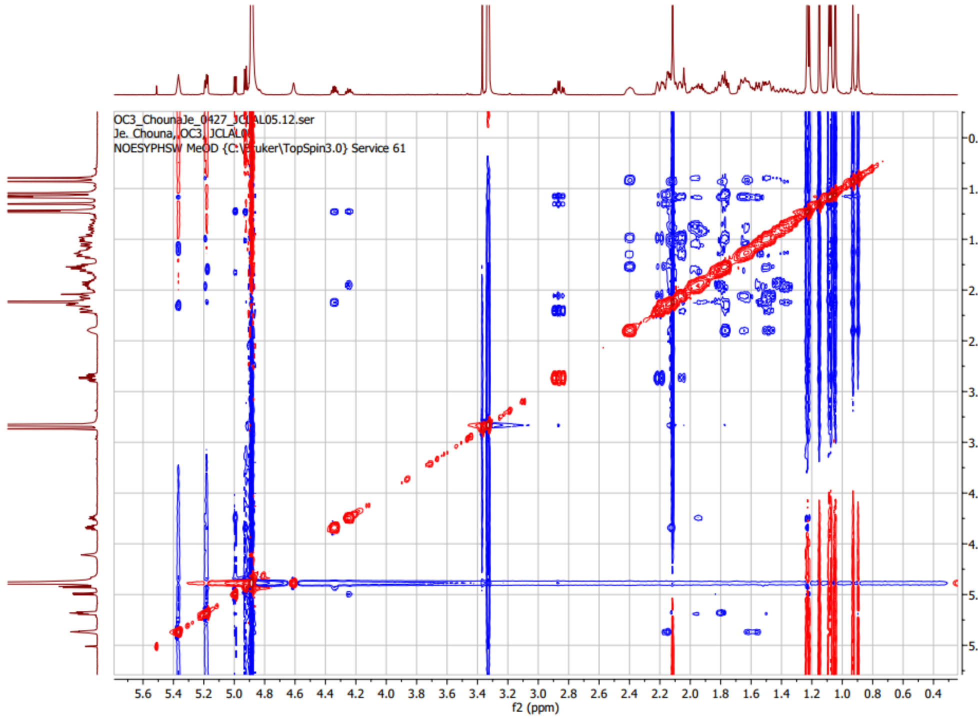

Figure S7. ^{1}H-^{1}H NOESY spectrum of compound **1**

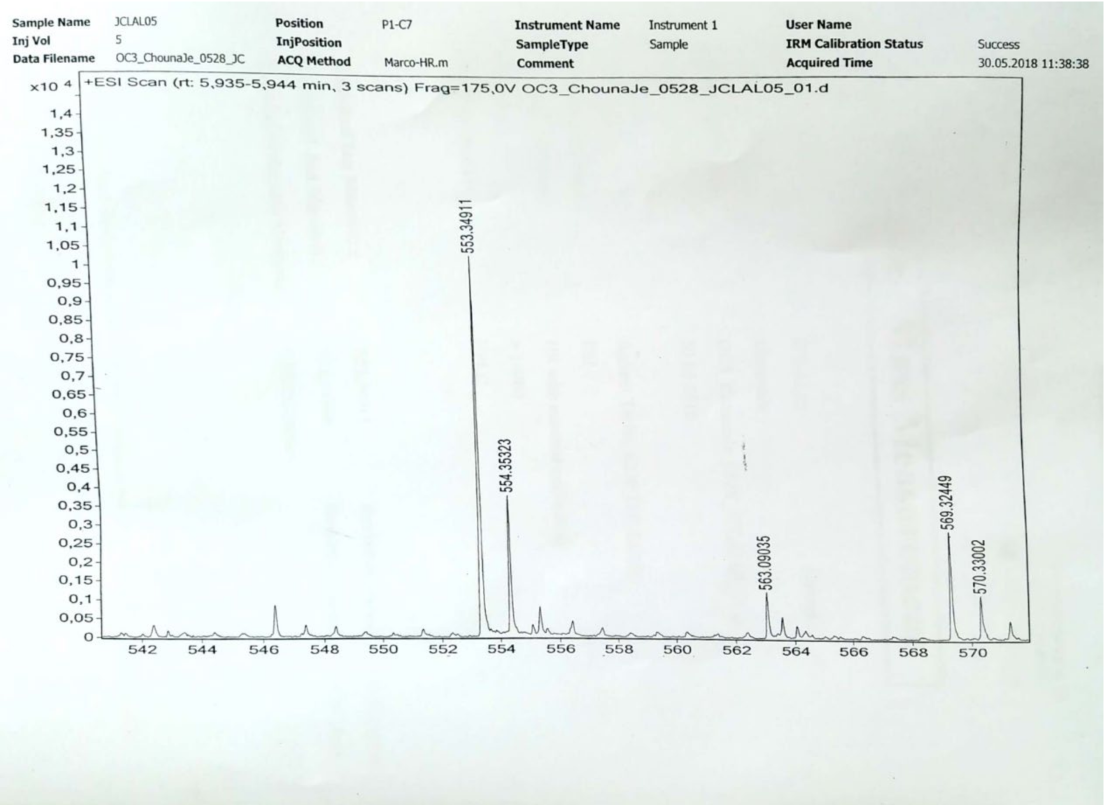

Figure S8. HRESIMS spectrum of $[M+Na]^+$ ion of compound **1**

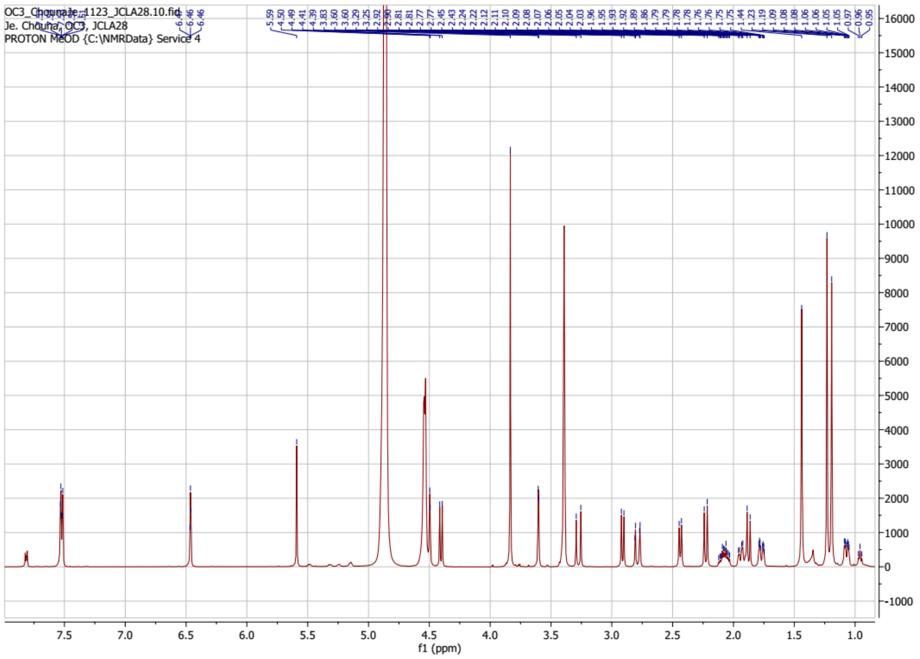

Figure S9. ^{1}H NMR spectrum of compound **2**

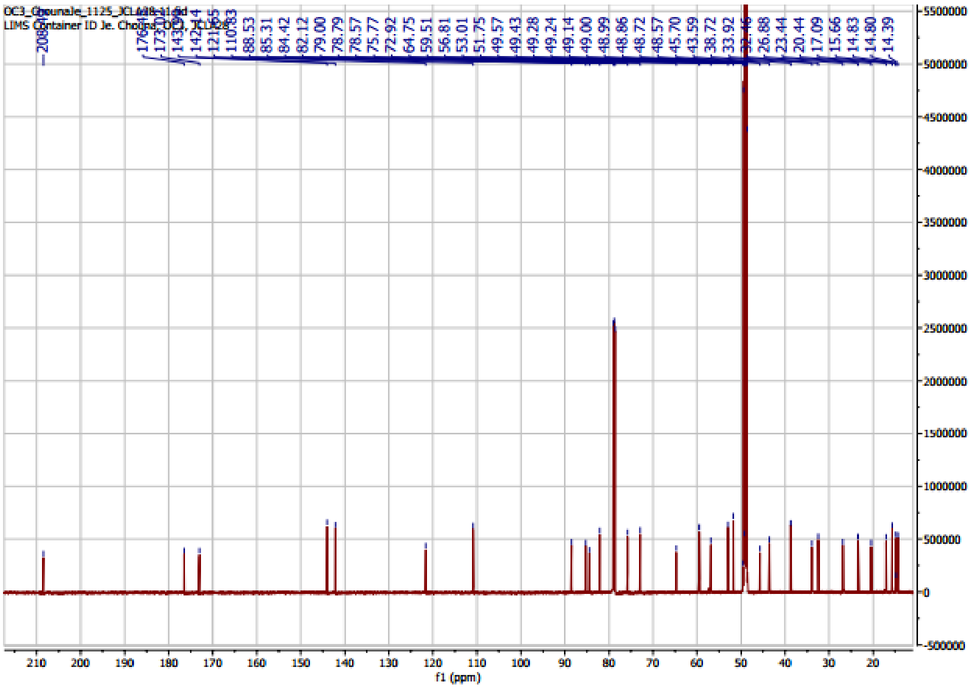

Figure S10. ^{13}C NMR spectrum of compound **2**

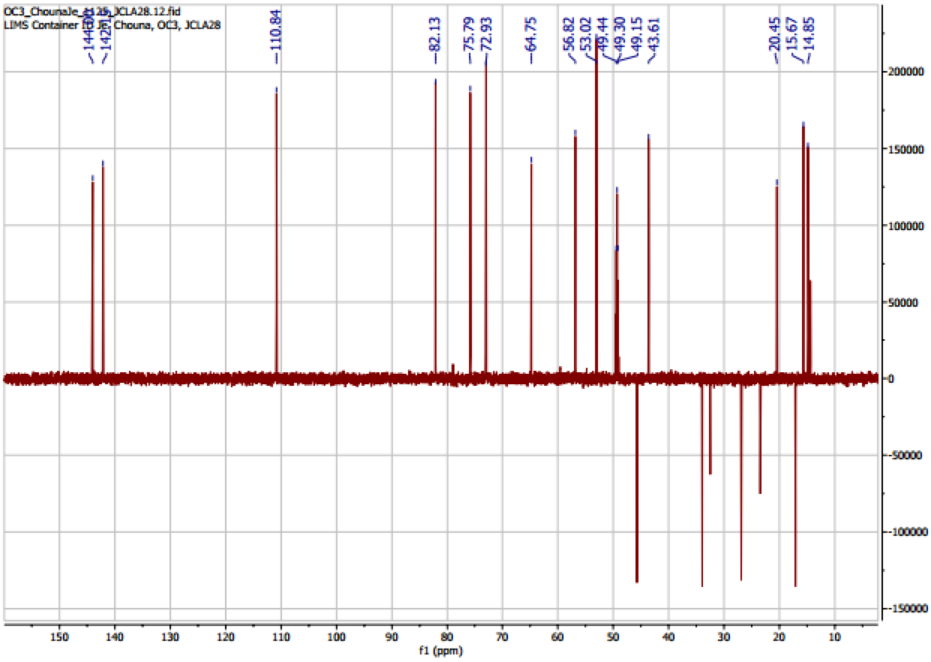

Figure S11. DEPT 135° spectra of compound **2**

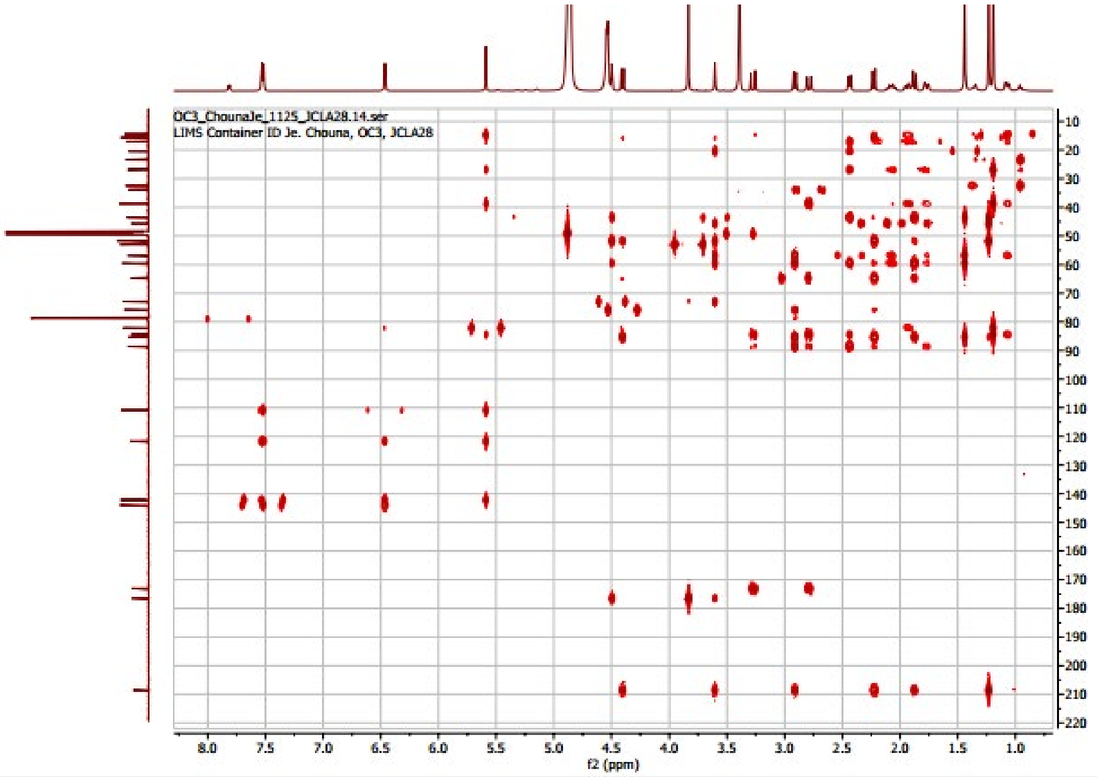

Figure S12. HMBC spectrum of compound **2**

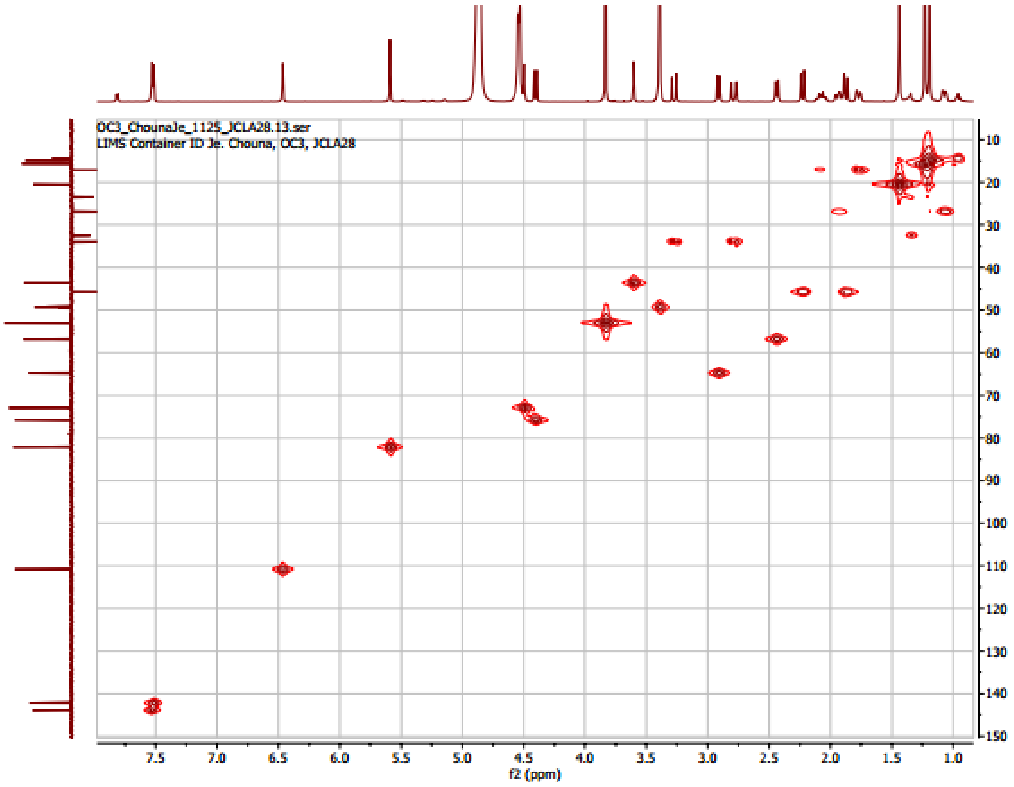

Figure S13. HSQC spectrum of compound **2**

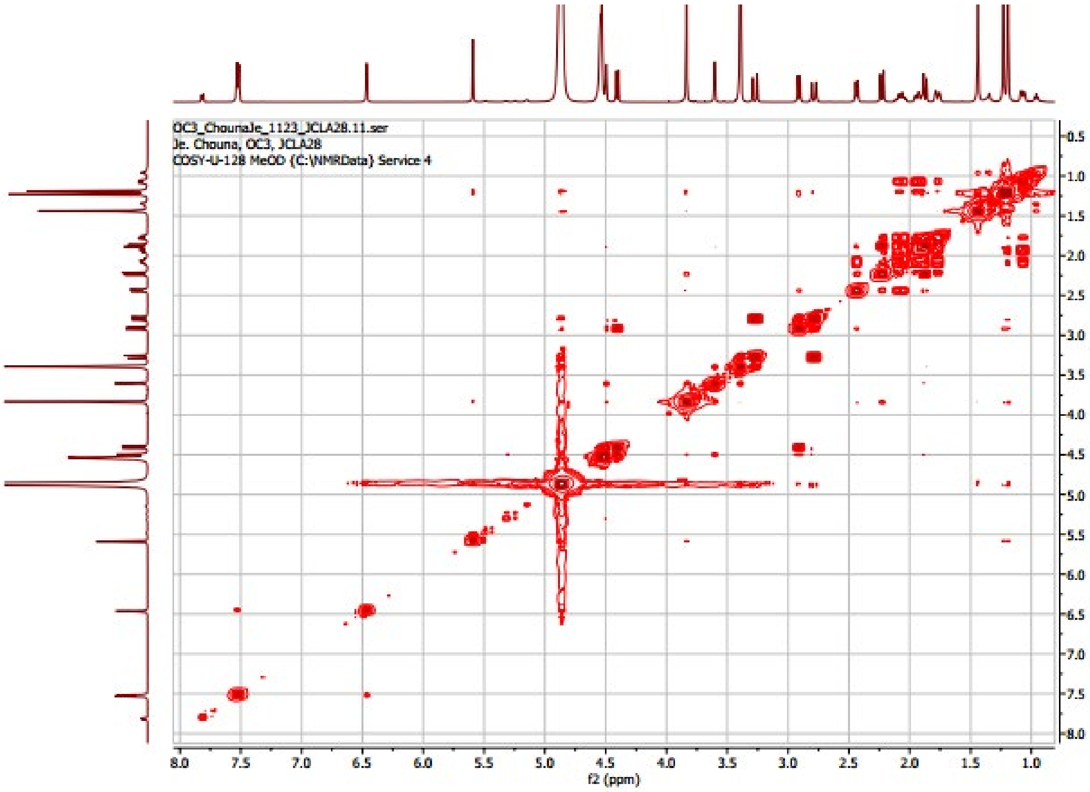

Figure S14. ^{1}H-^{1}H COSY spectrum of compound **2**

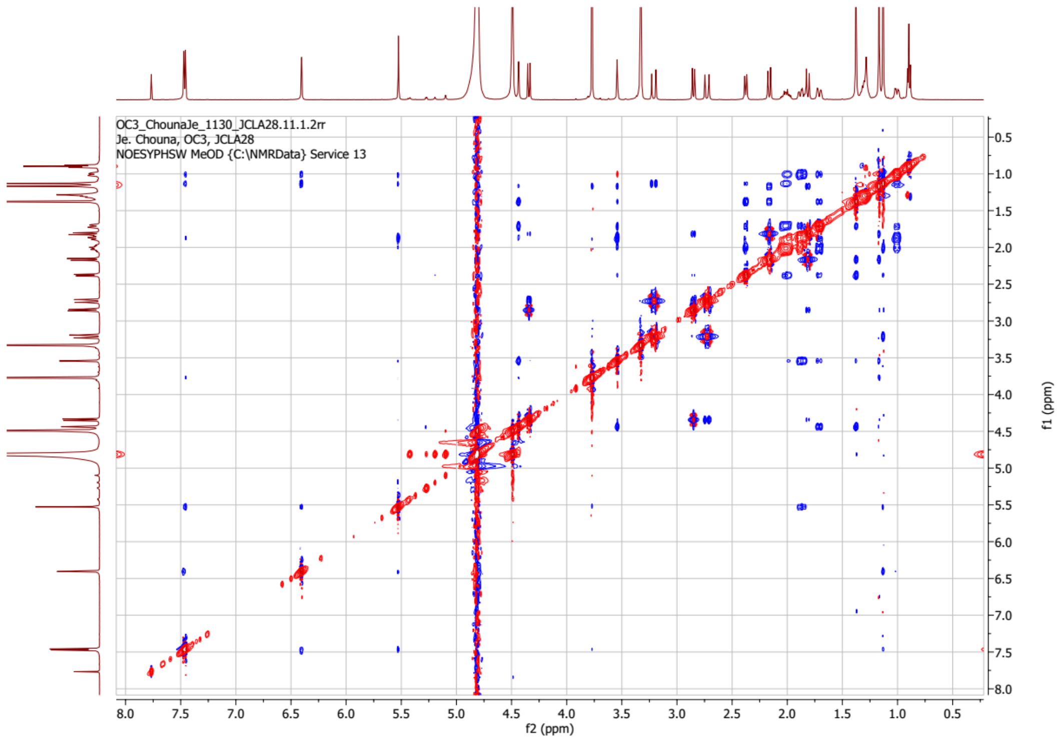

Figure S15. NOESY spectrum of compound **2**

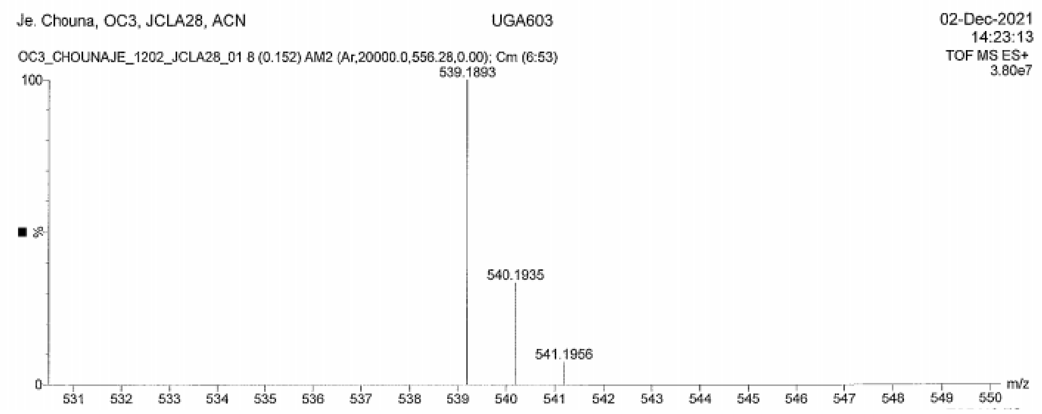

Figure S16. HRESIMS spectrum of $[M+H]^+$ ion of compound **2**
